# Analyzing the vulnerabilities in Split Federated Learning: assessing the robustness against data poisoning attacks

**DOI:** 10.1038/s41598-025-15993-8

**Published:** 2025-08-19

**Authors:** Aysha-Thahsin Zahir-Ismail, Raj Shukla

**Affiliations:** Computing and Information Science, Anglia Ruskin University, Cambridge, UK

**Keywords:** Federated Learning, Split Federated Learning, Data poisoning, Privacy, Computational science, Computer science

## Abstract

Distributed Collaborative Machine Learning (DCML) offers a promising alternative to address privacy concerns in centralized machine learning. Split learning (SL) and Federated Learning (FL) are two effective learning approaches within DCML. Recently, there has been growing interest in Split Federated Learning (SFL), which combines elements of both FL and SL. This research provides a comprehensive study, analysis, and presentation of the impact of data poisoning attacks on Split Federated Learning (SFL). We propose three attack strategies: untargeted attacks, targeted attacks, and distance-based attacks. All these strategies aim to degrade the performance of the DCML classifier. We evaluate the proposed attack strategies using two case studies: Electrocardiogram Signal Classification and Automatic Handwritten Digit Recognition (MNIST dataset). We conducted a series of attack experiments, varying the percentage of malicious clients and the model split layer between the clients and the server. A comprehensive analysis of the attack strategies reveals that distance-based and untargeted poisoning attacks have a greater impact on evading classifier outcomes compared to targeted attacks in SFL.

## Introduction

Artificial intelligence (AI) and machine learning (ML) are widely deployed by organizations of all sizes, from governments and tech giants to small online retailers. In the tech industry, a staggering 83% leverage AI-powered technologies for application development. Machine learning, with its ability to significantly improve productivity and performance, brings efficiency to various domains, including product recommendation, biomedical image classification, computer vision, and natural language processing^1–3^.

Most machine learning applications rely on supervised learning models^2^. The performance of these models in real-world scenarios hinges on the quality of the training data^4^. To achieve optimal model performance and accuracy, machine learning systems require a vast amount of high-quality training samples. However, obtaining labeled training data can be challenging. Additionally, centralizing all training data on a single server raises privacy concerns, especially when dealing with sensitive information. Regulations like the General Data Protection Regulation (GDPR) must be considered when aggregating private data. Distributed Collaborative Machine Learning (DCML) offers a potential solution. This approach enables multiple participants to collaboratively train a shared global model while keeping their training data local^5^. In essence, DCML provides collaborative ML architectures that train models using shared training data in distributed environments.

Federated learning (FL) and split learning (SL) are two DCML approaches that address privacy concerns in centralized machine learning^6,7^. In FL, multiple clients train a complete machine learning model using their local data. The locally trained models from all clients are then aggregated to create a global model at the server. While FL prevents sharing raw data, it might not be suitable for clients with limited computing resources for handling large models. Additionally, both the server and clients have access to local and global models, raising potential privacy issues for client training data and server model parameters. Communication delays, heterogeneous systems in distributed learning environments, and data dynamism pose further challenges in FL with multiple clients.

Split learning (SL) was introduced to address these limitations. By splitting the machine learning model between the client and the server, SL mitigates resource constraints and enhances model privacy. In SL, the client and server only have access to their respective portions of the split model^8^. However, SL is less efficient with a large number of clients, as it can only train one client at a time. This idling of other clients leads to longer training times^9^.

Split Federated Learning (SFL) emerges as an advanced DCML approach that addresses limitations of both FL and SL. SFL adopts a hybrid architecture. Similar to SL, the model is split, enabling clients with limited resources, like low-power IoT devices, to participate in training. However, unlike SL, SFL incorporates parallel computation, inspired by FL, to mitigate the training overhead associated with a large number of clients^9^. This approach is particularly beneficial for resource-constrained devices commonly found in the Internet of Things (IoT). The effectiveness of SFL has been demonstrated in various applications like bioinformatics, IoT, and sequentially distributed data^10–13^.

While extensive research has evaluated the security of FL, revealing vulnerabilities to model poisoning attacks that manipulate gradients to intentionally decrease accuracy^14^, the security landscape of SFL remains largely unexplored. Existing studies primarily focus on FL, highlighting its susceptibility to inference attacks where attackers attempt to reconstruct private data from the client or server^15^. However, minimal research analyzes the potential vulnerabilities of SFL to adversarial attacks, particularly when training data is distributed across numerous clients.

This work examines how a malicious client can initiate data poisoning attacks in the SFL system. Data poisoning attacks attempt to manipulate training data that eventually influences the learning output of the trained model. Data poisoning attacks broadly take the form of clean label poisoning and dirty label poisoning, where the former injects tampered data into the training set and the latter manipulates the training labels, such as label flipping^16^.

Accordingly, the major contributions of this paper can be summarized as follows:

1. This research proposes targeted, untargeted, and distance-based data poisoning attacks on SFL to evade the aggregated model outcomes.
2. The research tests the proposed targeted, untargeted, and distance-based data poisoning attack strategies for two case studies: handwritten digit classification (standard MNIST dataset) and a novel application of ECG signal classification for arrhythmia heartbeat types using SFL.
3. This paper conducts an extensive study on the proposed attacking strategies on SFL, varying the proportion of model split and malicious client percentage in MNIST and healthcare ECG signal datasets.

The remainder of this paper is structured as follows. Section “Related work” provides a comprehensive overview of existing research relevant to the topic. Section "Methodology: data poisoning attacks in Split Federated Learning" delves into the proposed attack techniques designed specifically for Split Federated Learning (SFL). Section “Implementation” focuses on the implementation details, including the system architecture, employed datasets, and setup of a poisoning attack. Section "Results and discussion" outlines the obtained results and analyzes the performance of the poisoning attacks across the chosen case studies. Finally, Section "Conclusions and future work" summarizes the key findings and concludes the paper.

## Related work

The related work is divided on two sections. First we provide, the backgroung on Federated Learning, Split Learning, and SplitFed Learning. Subsequently, we will discuss the security aspects of these methods.

### Background

#### Federated Learning

**Fig. 1:**
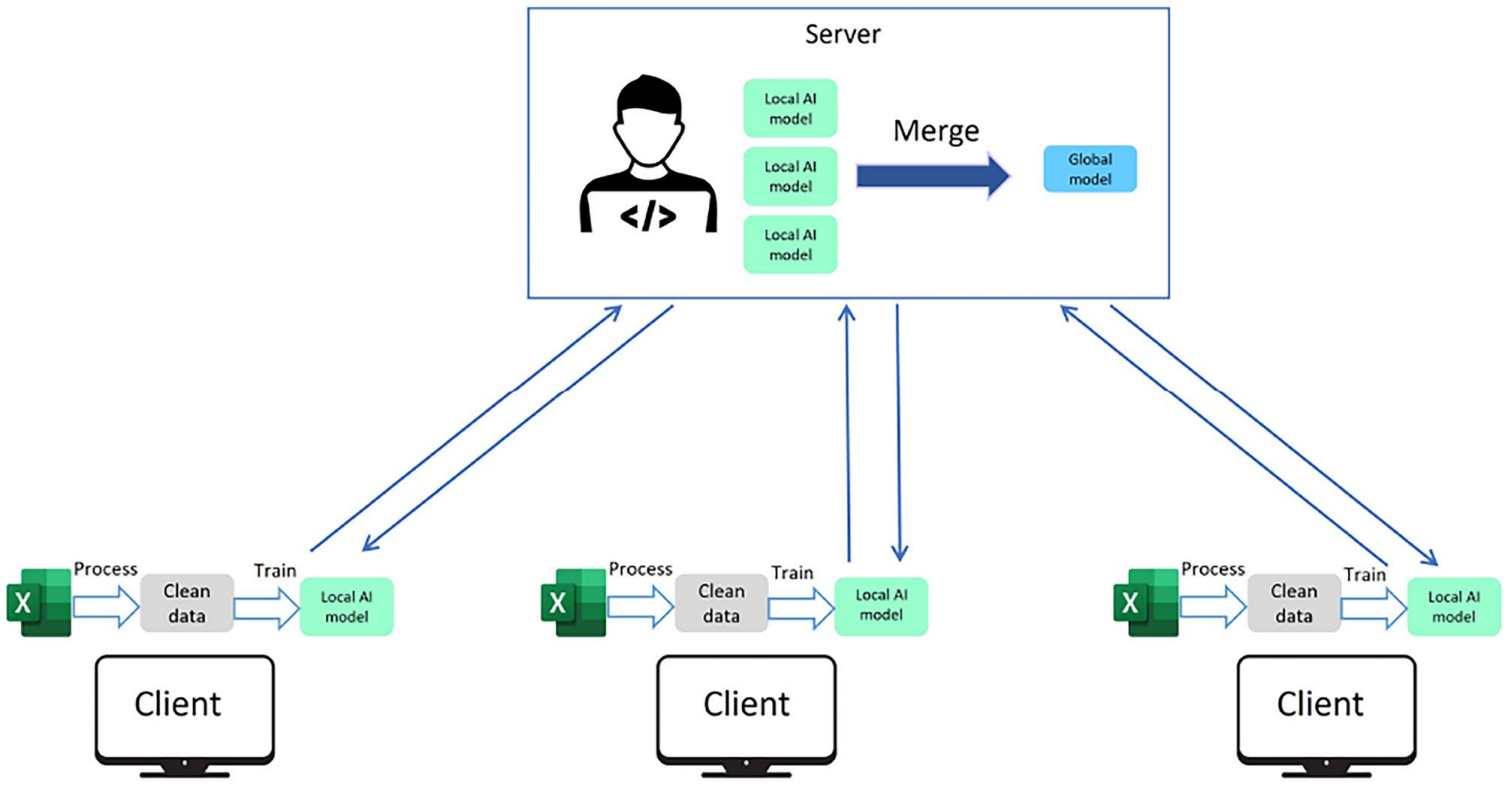
Federated Learning.

The basic architecture of Federated Learning (FL) is depicted in Fig. 1. The fundamental idea behind FL is the collaborative training of ML models among distributed data holders. In a decentralized setting with multiple clients, each client has its local data and trains the complete ML model. After each training iteration, all clients transfer the updated weights, obtained from computing forward and backward passes on their local models, to a central server. FedAvg, a commonly used aggregation algorithm, is employed by the server to achieve a global update for the ML model. This global update is then passed on to the clients for the subsequent iteration. Unlike traditional centralized ML, where raw client data is shared for training, FL only shares the model parameters with the server or other clients. As a result, FL reduces communication costs and eases the networking overhead involved in Internet of Things (IoT) services with various entities and limited resources^6^.

#### Split Learning

**Fig. 2:**
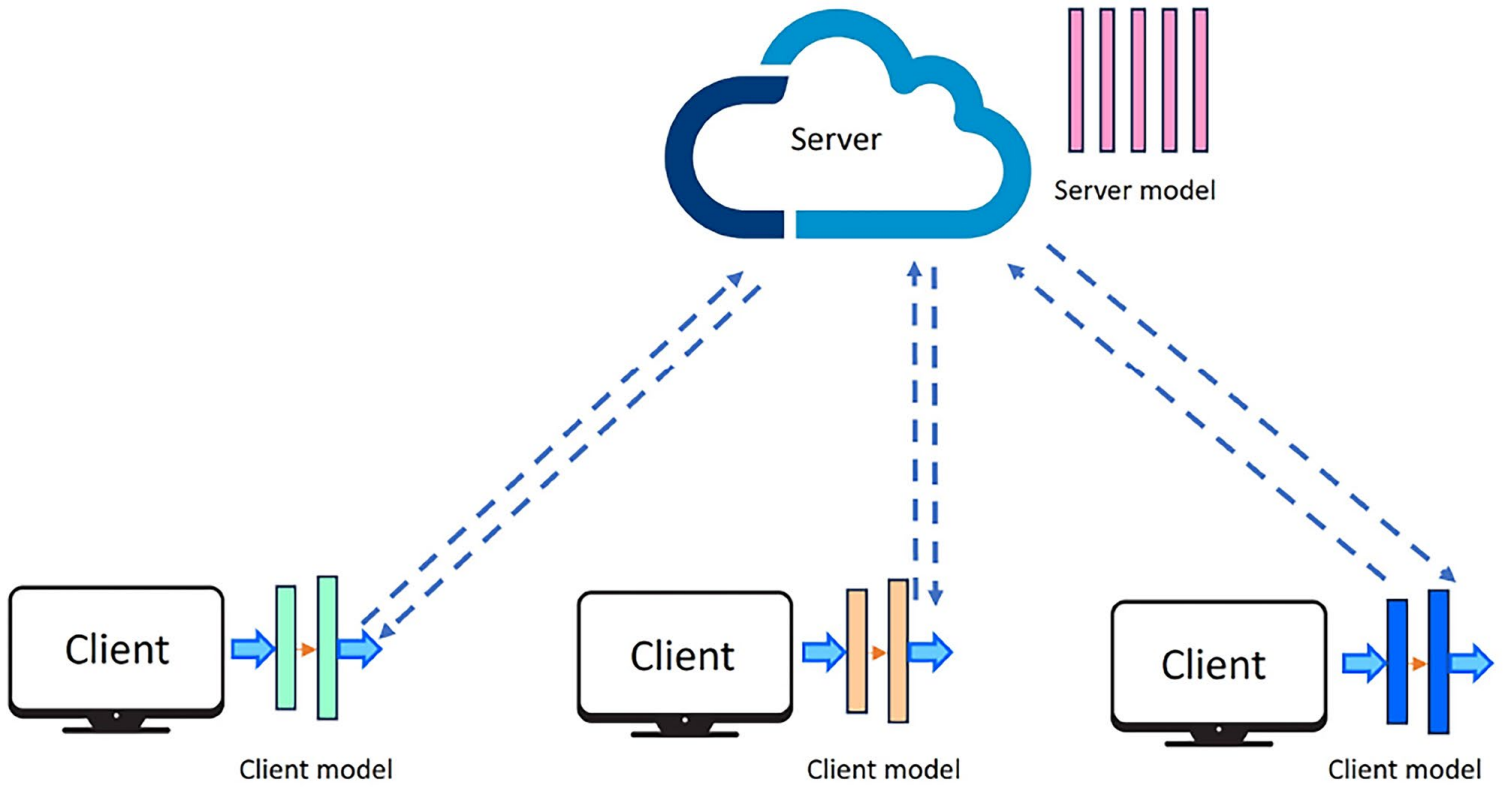
Split Learning.

Figure 2 presents the basic architecture of Split Learning (SL). SL^7^ is a DCML approach that divides the ML/DL model between the client and server. The model layer at which the split occurs is referred to as the cut layer, and the output generated is termed smashed data. Computations on initial model layers are performed by the client, and the later layers are handled by the server, thereby keeping local training data private, similar to FL.

In SL, sequential training is performed where each client performs forward propagation with its own model segment until the *cut layer*, which is the last layer of the client-side model split. The *smashed data* (activations from the cut layer) is received by the server, which continues forward propagation with the model’s remaining layers^17^. Once the forward propagation is completed, the server determines the loss and begins backpropagation. The gradient calculated up to the cut layer is passed on to the client to continue its backpropagation. This entire process constitutes one training round, and the updates are then sent to the next client^17^.

#### Split Federated Learning

**Fig. 3:**
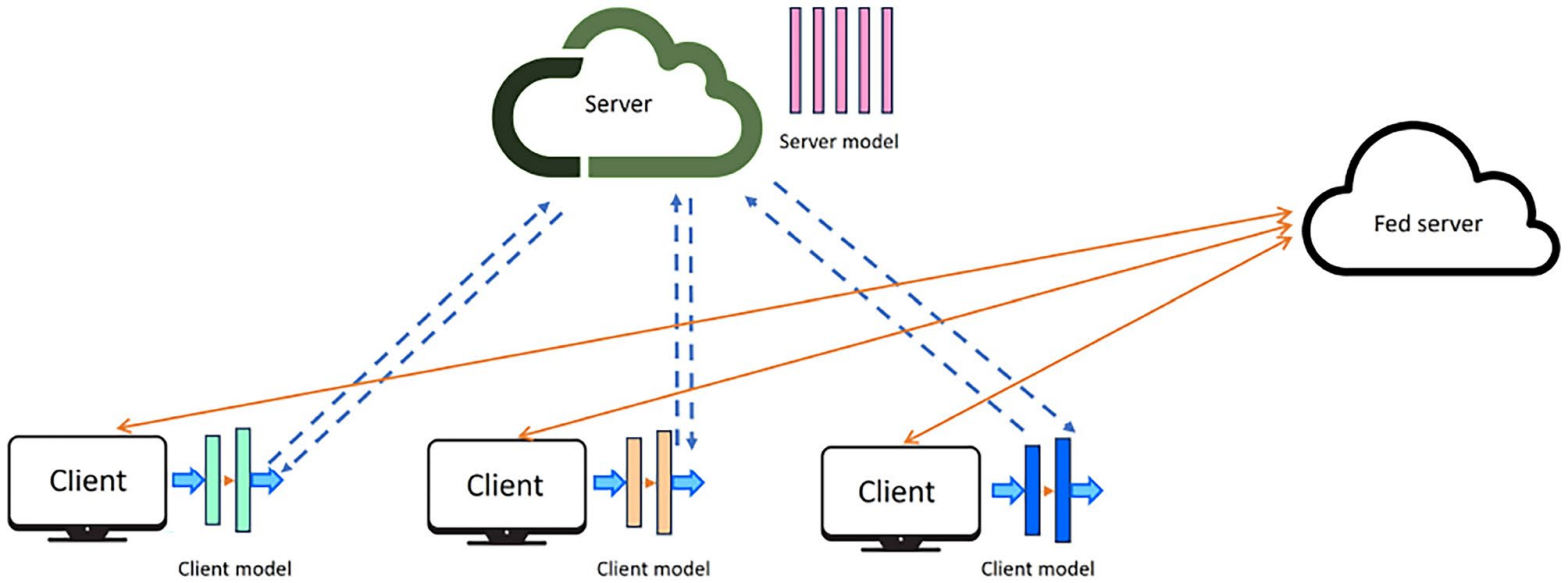
Split Federate Learning.

The basic architecture of Split Federated Learning SFL is represented in Fig. 3. SFL-based distributed client environments include a main server, a fed server, and a group of clients, as represented in the figure. The full model *N* is split into a client-side model $N^C$ and a server-side model $N^S$. At each global epoch, all clients interact with the server in parallel, and the main server aggregates the parameters to generate a global server-side model. The client model synchronization is carried out in parallel at the fed server. Considering *k* clients at time instance *t*, the client-side model of each client can be represented as $N_{k,t}^{C}$. The smashed data of each client at *t* is $S_{k,t}$. At $t=0$, each client *k* performs forward propagation of its model split and sends the activations $S_{k,t}$ along with the true labels to the server^9^.

Upon receiving the smashed data, the server performs forward propagation with its model split, computes the prediction, and calculates the loss using the actual labels and the predicted labels ${\hat{y}}$. Further, the server updates the global server-side model and propagates the gradient back to the client. Simultaneously, when each client receives the backpropagated smashed data from the server, it is sent to the fed server to aggregate and generate a global client-side model update, which is then sent back to all *k* clients^9^. Table 1 presents the basic notations used in SFL.

### Security in federated and split learning

There has been work done in the literature on the security of federated, split, and split federated learning. Federated learning is susceptible to numerous adversarial attacks, mainly poisoning attacks and information extractions^18^. Model poisoning and data poisoning attacks are the main security threats faced by FL. In data poisoning attacks, the attacker manipulates the labels or features of data samples before the training phase, leading the model to learn incorrect patterns. In contrast, model poisoning attacks target the machine learning model itself, manipulating local client updates rather than the data^19^. Typical backdoor attacks in FL exploit this vulnerability. As classified by Lyu et al. (2020), malicious actors in FL can be categorized into three groups: malicious servers, insiders, and outsiders. Poisoning attacks by untrusted participants can significantly degrade the performance of FL^20^. Additionally, the security of FL is challenged by inference attacks induced by compromised servers. These servers can potentially learn and extract private client data using the gradient updates they receive^21^.

Darzi et al. have discussed the adversarial attack on FL for medical images and have shown that these attacks are transferable. They have shown that the attack applied on one model can also be applied to other models^22^. Tekli et al. have provided the method where deep learning-assisted attacks can cause re-identification of obfuscated images, and such attacks can be amplified by adversaries while working collaboratively in an FL setup^23^. Guo et al. have shown the image-based fairness data poisoning attacks that can lead to compromised AI models in an FL setup. They also propose a defense mechanism using a GAN-based neural network to counter such attacks for image data^24^. Murmu et al. have proposed a GAN-based model to enhance the reliability of the FL algorithm. The technique proposed in the paper adapts each data sampling to the local target while performing optimization. They tested the approach using various colored image datasets^25^. Garcia et al. have proposed a blockchain-based approach to make FL secure against adversarial attacks. They enhanced the robustness against backdoor and Byzantine attacks in FL and have also tested their method for image datasets^26^.

Similar to Federated Learning (FL), SL faces the challenge of model stealing, as demonstrated by Erdoğan et al.’s attack causing client model inversion^27^. This is particularly concerning in two-party Split Learning, where private label leakage attacks can occur when unauthorized parties attempt to infer client labels (e.g., Li et al.’s gradient analysis attack^28^). To address these privacy threats, researchers have proposed various techniques like distance correlation and differential privacy^29,30^. Combining FL and SL offers a promising approach by leveraging their strengths while mitigating individual limitations.

SFL is a hybrid DCML architecture that combines features of both FL and SL. It integrates the parallel training/testing of client-side models, as seen in FL, with the model split between client and server, as performed in SL. The SFL system consists of client and server segments, along with an additional server called the fed server on the client side. The fed server performs the FedAvg aggregation algorithm on updates provided by the client and is responsible for synchronizing the global model updates of multiple clients^9^.

Each client in SFL performs forward propagation on the client-side model split with their local training data until the cut layer. The server then proceeds with forward and backward propagation, as in SL, and sends the updated gradients to all its clients in parallel. Further, each client completes the backward pass on their client-side model, and updates are forwarded to the fed server. The fed server conducts FedAvg on the updates from all clients, resulting in a client-side global model with the parameters redirected to all clients^9^.

SFL effectively addresses the difficulties encountered in both FL and SL, providing greater privacy than FL. However, there is a significant risk of data poisoning attacks during collaborative training among distributed clients and a server with SFL. Malicious participants can introduce poisonous data during the training process, which is difficult for the aggregator to detect.

Motivated by this analysis, this research introduces data poisoning attacks against SFL and aims to fill the research gap in studying the robustness of SFL. This paper proposes targeted and untargeted attacks, along with a novel distance-based attack strategy, and performs a comparative analysis of these attacks on the MNIST and healthcare ECG signal datasets.

**Table 1:** Principle Notations of SFL.

**Symbol**	**Definition**
*N*	The full deep learning model
$N^C$	Client-side model split
$N^S$	Server-side model split
$S_{k,t}$	Smashed data for client k at time t
*y*,${\hat{y}}$	Actual labels and predicted labels respectively

## Methodology: data poisoning attacks in Split Federated Learning

This section discusses the proposed methodology of data poisoning attacks on SFL. We discuss the threat model and algorithms used to attack SFL-based DCML.

### Proposed threat model in split federated system

In SFL, participating clients share the smashed data with the server segment of the model, ensuring the privacy of the client-training data. Consequently, none of the functional components in the framework verify the quality and security of the training data. Due to the introduction of these vulnerabilities, i.e., privacy and security of client-training data, the server with the split of the global model is now prone to data poisoning attacks from malicious clients within the client group.

This paper’s threat scenario considers the presence of a subgroup of malicious participants or a percentage of participants who are either malicious or under the control of a malicious adversary. The main objective of the malicious client or adversary is to poison the training data and compromise the training efficiency. This is carried out by manipulating the training through label perturbation.

**Fig. 4:**
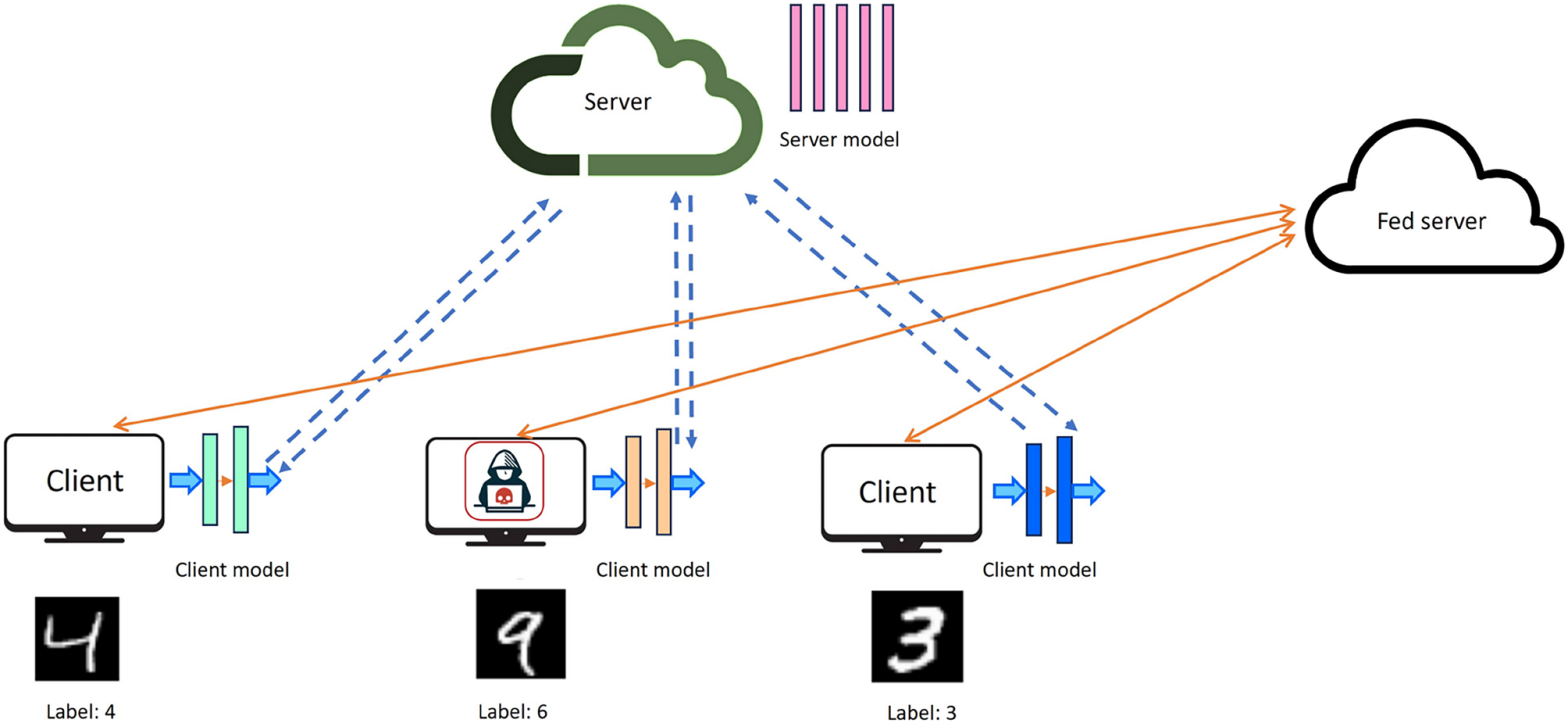
Threat Model.

Figure 4 illustrates the data poisoning attacks by label flipping in the SFL model. In this scenario, one malicious client perturbs the label “9” of the private training sample to the label “6,” thereby infecting the local model.

In this paper, the training data is perturbed using novel targeted, untargeted, and distance-based label-flipping attack algorithms that evade the classifier to produce incorrect results. This work considers the following realistic assumptions for data poisoning attacks:

1. This paper considers a realistic scenario where only a percentage of clients are malicious or controlled by an external adversary. Given a group of $X$ clients, the adversary can control $y$% of the $X$ clients. In this research, we evaluate the performance of SFL under varying percentages of malicious clients, reflecting the practicalities of real-time distributed learning scenarios.
2. The paper assumes a realistic scenario where each malicious client can only manipulate its private training data. The adversary or the malicious client cannot influence the aggregation operation of the federated server to produce a global client-side update and does not have access to the benign participants’ training data.
3. This paper assumes an honest main server. The assumption of an honest main server is similar to studies that conducted client-side inference attacks^15^.
4. The attack model presented in this paper assumes that the attacker is agnostic to the model. The attacker is not aware of the specifics of the model architecture or parameters used but relies solely on flipping the labels of the training dataset. Due to the nature of such attacks, they are highly transferable; that is, an attack performed on one model remains effective on other, more complex models as well^31,32^.

### Proposed data poisoning attack strategies

In this work, the data poisoning attack strategy is implemented by perturbing the labels of the dataset, i.e., by changing the class labels, which ultimately causes the trained model to generate incorrect predictions. In SFL, malicious clients or adversaries train the client-side model with altered labels (poisoned training data) and transmit the model parameters to the server. Since the client models are trained on poisoned data, the server-side model also becomes corrupted, resulting in decreased performance.

Suppose that the given classification task contains a list of *L* training labels. Out of these labels, some labels *l*, where $l \in L$, are maliciously replaced by label $l' \in L$. Considering this scenario, we introduce three attack types in this paper that replace the correct labels in the dataset with incorrect ones.

#### Targeted poisoning attacks

Targeted poisoning attacks replace the labels in the dataset with specific incorrect labels. The main motivation of the attacker is to affect the predictions of the replaced label. For example, in a recommendation system, the labels may be altered to influence users not to purchase items from specific companies. This manipulation impacts the AI model unfairly, causing it to not recommend products from specific companies, thus benefiting the adversary which may be a competitor.

Thus, in the targeted attacks, the adversary selects the labels *l* of the source class $S_c$ and replaces them with labels *l’* of a target class $T_c$, where *(l, l’)* $\in$ *L*. Here, only the label of $S_c$ is manipulated, and the remaining class labels remain the same. Targeted poisoning attacks aim to reduce the accuracy of the classifier for the targeted source class while not affecting the accuracy of the remaining non-targeted samples. Algorithm 1 represents the scenario of the targeted poisoning attacks on SFL.

**Algorithm 1:**
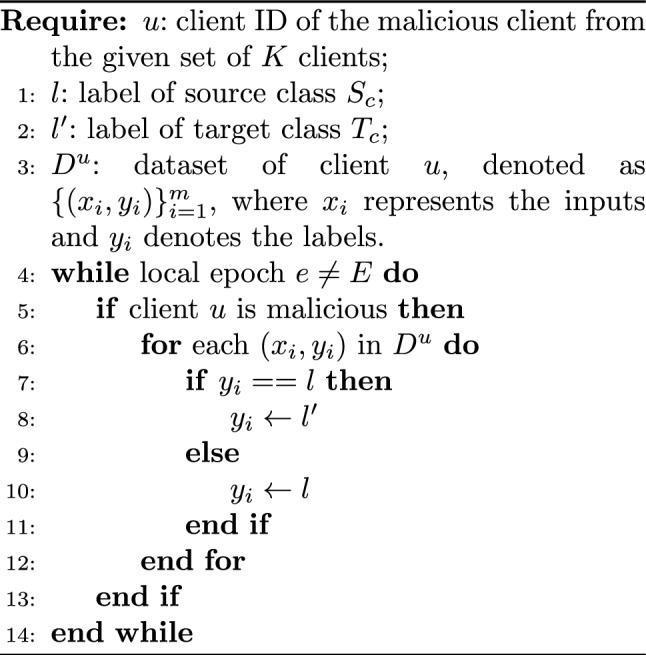
Targeted Poisoning Attack

**Algorithm 2:**
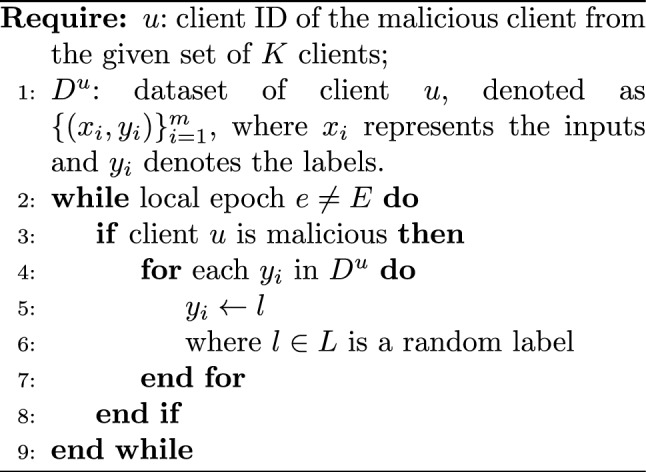
Untargeted Poisoning Attack

#### Untargeted poisoning attacks

In an untargeted attack, the labels are randomly perturbed with incorrect labels. The intuition behind an untargeted attack is that it reduces the accuracy of the model without prioritizing any specific class. For example, in an application that converts numbers in an image to their actual numerical values, all digits are equally important. In such a scenario, the attacker will randomly flip the labels of the images to poison the data. This affects the performance and reduces the overall accuracy of the model.

Thus, the proposed untargeted attacks do not target the label of a specific source class. Instead, they randomly flip a selected set of labels *l* with *l’*, where *(l, l’)* $\in$ *L*. Untargeted attacks can also flip all class labels to one random class label, drastically reducing the accuracy of the classifier. Initiated by a set of malicious participants, untargeted attacks have a greater impact compared to targeted attacks due to the iterative submission of malicious parameters to the server. Algorithm 2 depicts the untargeted poisoning attack. Untargeted attacks attempt to degrade the performance of the classifier as a whole rather than the accuracy of a specific class.

#### Distance-based poisoning attacks

In a distance-based attack, the attacker aims to replace a label with another label of reverse polarity. The motivation for such an attack is that prominent labels in the dataset can be altered to their completely opposite labels. For example, consider a binary classification problem with two labels: “disease has occurred” and “disease has not occurred.” The significant label here is “disease has occurred.” The attacker will replace instances where “disease has occurred” with “disease has not occurred,” causing the model to make incorrect predictions for these samples. Altering the specific label “disease has occurred” to its reverse, “disease has not occurred”, would cause a more severe adverse impact on the model.

**Algorithm 3:**
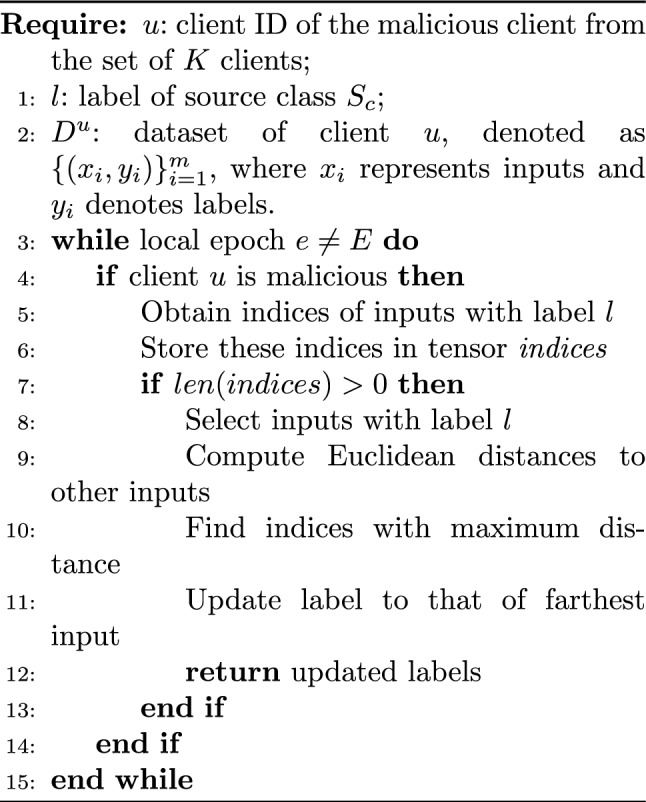
Distance-based Poisoning Attack

Thus, in the proposed distance-based poisoning attacks, the adversary optimizes and improves the efficiency of targeted attacks by carefully selecting the target class $T_c$. The label of the selected $T_c$ is used to replace the labels of the source class $S_c$. To implement a distance-based attack, the adversary initially selects a source class $S_c$ with the label *l* and calculates the Euclidean distance between samples of $S_c$ and other training samples where *l* and *l’* are not equal. The Euclidean distance computes the distance between input samples, provided the input samples are real-valued vectors.

Next, the training sample with the maximum distance is selected, and its corresponding class is chosen as the target class $T_c$. The label of this selected target class is used to poison the training samples of $S_c$. Using the maximum distance increases the success rate of the attack. When the source class label is replaced with the label of the sample having the maximum distance, the impact of the poisoning attack is increased.

In a multi-class classification problem, the adversary can select different source classes $S_{c}$ to initiate attacks by computing the Euclidean distance between input images and identifying the target class $T_{c}$ that is farthest away. For example, suppose the input image belongs to class ’1’, and its true label is also ’1’. The algorithm calculates the Euclidean distance from this image to all other images in the batch. If, for instance, the image labeled ’5’ is the farthest from the image labeled ’1’ in terms of Euclidean distance, then the label of the image ’1’ is replaced with ’5’ to create an adversarial target. The Euclidean distance is computed as the square root of the sum of the squared differences between corresponding pixel values of the two images. Algorithm 3 represents distance-based data poisoning attacks that enhance the risk to the ML model since they have a higher impact than targeted poisoning attacks. This type of attack represents a potential threat to SFL in real-world applications.

## Implementation

This section discusses the implementation details of the proposed attack methods. We describe the datasets involved used for the research, the model architecture used for training, the experiment setup for SFL, and data poisoning attacks.

### Dataset

We test the proposed methodology using two differnt case studies as mentioned below.

#### Case study 1 - automatic handwritten digit recognition (MNIST dataset)

The MNIST dataset is a benchmark dataset for machine learning and deep learning classifiers, comprising handwritten digits. It consists of 60,000 grayscale images for training and 10,000 grayscale images for testing, where the images are classified into 10 different classes labeled as ’0’ to ’9’. Each image in the dataset is of size 28x28 pixels, totaling 784 features in each image^33^. We chose MNIST dataset as it is renowned and a challenging benchmark dataset in machine learning and has been extensively utilized by researchers.

#### Case study 2 - ECG signal classification (ECG dataset)

Automatic classification of ECG signals to detect arrhythmia types eliminates the need for manual signal analysis by physicians and facilitates easy monitoring of heart conditions. For this application, the MIT-BIH Arrhythmia dataset is utilized, which contains ECG signals used to classify arrhythmia heartbeat types^34^. This standard database comprises 48 records, with each record containing ECG signals obtained from two separate channels. Each record spans 30 minutes, selected from a 24-hour period. In this study, 26,490 samples were gathered. The collected samples represent 5 different classes of heartbeat types provided in Table 2. Of the total samples half of them are selected randomly to train the model and the remaining are used for the testing process. ECG dataset was chosen because it pertains to healthcare, where privacy is a critical concern due to the presence of sensitive patient data. As our method focuses on privacy-preserving techniques, we have opted for this dataset.

**Table 2:** Arrhythmia Heartbeat Types.

**Class Label**	**Type of Heartbeat**
N	Normal beat
L	Left bundle branch block
R	Right bundle branch block
A	Atrial premature contraction
V	Ventricular premature contraction

### Model architecture

We utilize a 1-Dimensional fully connected dense neural network for the MNIST datasets, and a convolutional neural network (1D-CNN) for the ECG dataset. Table 3 presents the model architectures, which include four convolutional layers with ReLU activation functions, two max-pooling layers, two fully connected dense layers, and a SoftMax activation function for classifying outputs into five categories of arrhythmia heartbeat types.

For training on the MNIST datasets, a deep feed-forward network is employed, consisting of an input layer followed by 10 dense layers. The final classification layer uses a ReLU activation function to classify input samples into one of the 10 classes in the dataset.

The input layer in the deep feed-forward network is analogous to the input layer in the 1D-CNN, receiving input samples from the dataset. In this work, the input size for the MNIST dataset is 784 (as the images are 28x28 pixels).

**Table 3:** Models and Datasets.

**Dataset**	**Model**	**No: of Labels**	**Size of Input**
ECG dataset	1D-CNN 4 Convolutional Layers 2 Dense Layers	5	124
MNIST dataset	Feed-forward network 10 dense layers	10	784

### SFL setup

For the MNIST datasets in the SFL scenario, there is one server and ten clients. The dataset consists of 60,000 training images, which are evenly partitioned among the ten clients. Thus, each client receives 5,000 training records and retains 1,000 records for validation and testing. The remaining 10,000 test images are unseen and reserved solely for evaluation purposes.

In the case of the ECG dataset, the SFL setup includes one server and five clients. Each client receives distinct and equal batches of data from the training set. The test set data is entirely excluded from training and reserved exclusively for evaluating model performance. The total number of training epochs is set to 50, as the model shows convergence within fewer rounds than 50 training epochs.

### Data poisoning attack setup

In this paper, data poisoning attacks are introduced where only *y*% of *X* clients are malicious or controlled by an external adversary. Specifically, we tested when *y* is 20% and 40%. The proposed targeted, untargeted, and distance-based attacks vary in terms of the percentage of malicious clients assigned for both datasets. In untargeted attacks, all labels of the malicious clients are manipulated and replaced with a class label that achieves the highest test accuracy in the SFL system. Specifically, we tested when *y* is for 20% and 40%.

In the case of targeted and distance-based attacks, the selection of source class $S_c$ depends on the success of the poisoning attack. In the SFL setting, compromised clients have access to global client-side model updates from the fed server. The malicious client can initiate a poisoning attack for different source classes in a multi-class classification problem and evaluate the impact of the attack that degrades the performance of the classifier.

In the proposed targeted poisoning attack, the source class $S_c$ is selected as the class that has the highest percentage of correctly identified samples by the classifier. By manipulating the labels of that class with the target class $T_c$ that has the second highest percentage of correctly classified samples. The source class $S_c$ is chosen for distance-based poisoning attacks in an analogous way to targeted attacks. Euclidean distance is computed between inputs that have the label as source class $S_c$ and other inputs. After measuring the distance, the input that has the label as source class $S_c$ is replaced with the label of the input that has a maximum distance.

In order to increase the impact of the attack, experiments were carried out with different model splits between the client and the server. In the 1D-CNN model for the ECG dataset, the model was split in two positions. At first, the model was split at the second convolutional layer forming two layers for the client segment and four layers for the server segment. Secondly, the model splits at the third convolutional layer forming three layers for the client and three layers for the server segment. The first and the second model splits are called ECGv1 and ECGv2 respectively. Similarly, the deep feed-forward network for the MNIST dataset was also split at two positions, at the second dense layer termed MNISTv1 and at the fourth dense layer referred to as MNISTv2. In the former split, the first two layers form the client-side model, and the remaining eight dense layers belong to the server segment. In the latter split, there will be four layers on the client-side model and six layers on the server-side model.

**Table 4:** Accuracy drop under different attack methods.

	Percentage of malicious clients	Untargetted attack		Targetted attack		Distance-based attack	
		*A*	$A_{d}$	*A*	$A_{d}$	*A*	$A_{d}$
MNISTv1	0	96.46	0	96.46	0	96.46	0
	20	95.92	0.56	96.13	0.34	06.09	0.38
	40	89.86	7.08	91.35	5.30	90.77	5.89
MNISTv2	0	96.54	0	96.54	0	96.54	0
	20	95.54	0.62	94.89	1.71	95.37	1.21
	40	86.06	11.48	90.58	6.17	88.56	8.26
ECGv1	0	88.87	0	88.87	0	88.87	0
	20	86.99	2.12	88.23	0.72	87.99	0.99
	40	33.87	61.89	83.19	6.39	76.67	11.48
ECGv2	0	88.89	0	88.89	0	88.89	0
	20	75	15.62	87.42	1.65	79.77	10.26
	40	26.50	71.31	82.62	7.05	75.46	15.11

## Results and discussion

This section examines the effect of data poisoning attacks on the MNIST and ECG datasets and the impact of varying the cut layers.

### Effects of data poisoning attacks

This section describes the results of data poisoning attacks on two independent case studies. The effects of targeted, untargeted, and novel distance-based poisoning attacks were examined for each of them.

Table 4 presents the accuracy and accuracy drop ($A_{d}$) in percentage for the two case studies and under different percentages of malicious clients. As seen in the table, the model’s accuracy is greatly reduced due to the untargeted poisoning attack. In the presence of a maximum number of malicious clients, the accuracy drops from 88.87% to 33.87%, resulting in a 61.89% drop in accuracy for ECGv1. For ECGv2, the success of the attack is even higher, resulting in a 71.31% depletion in accuracy. In ECGv2, it is observable that a small percentage of malicious clients can drastically reduce the accuracy of the model. Thus, the accuracy for MNISTv1 decreased from 96.46% to 89.86%. For MNISTv2, a bigger variance in accuracy is seen. When there are 20% malicious clients present, accuracy falls to 86.06%.

The success of targeted attacks is low compared to untargeted attacks. This is due to minimal perturbation in the training samples. The training data of malicious clients contain less corrupt data compared to untargeted scenarios, causing the accuracy to drop by no more than 7% in either of the split versions.

The accuracy after distance-based attacks is worse than targeted attacks, causing accuracy to drop up to 11.48% in ECGv1 and 15.11% in ECGv2. However, the overall accuracy depletion is more for distance-based attacks compared to targeted attacks. Similar to distance-based attacks induced in the ECG dataset, here the adversary targets a specific class. By manipulating class labels with distance measures, the maximum drop in accuracy is 5.89% in MNISTv1. In MNISTv2, the value of $A_{d}$ is 8.26%.

**Fig. 5:**
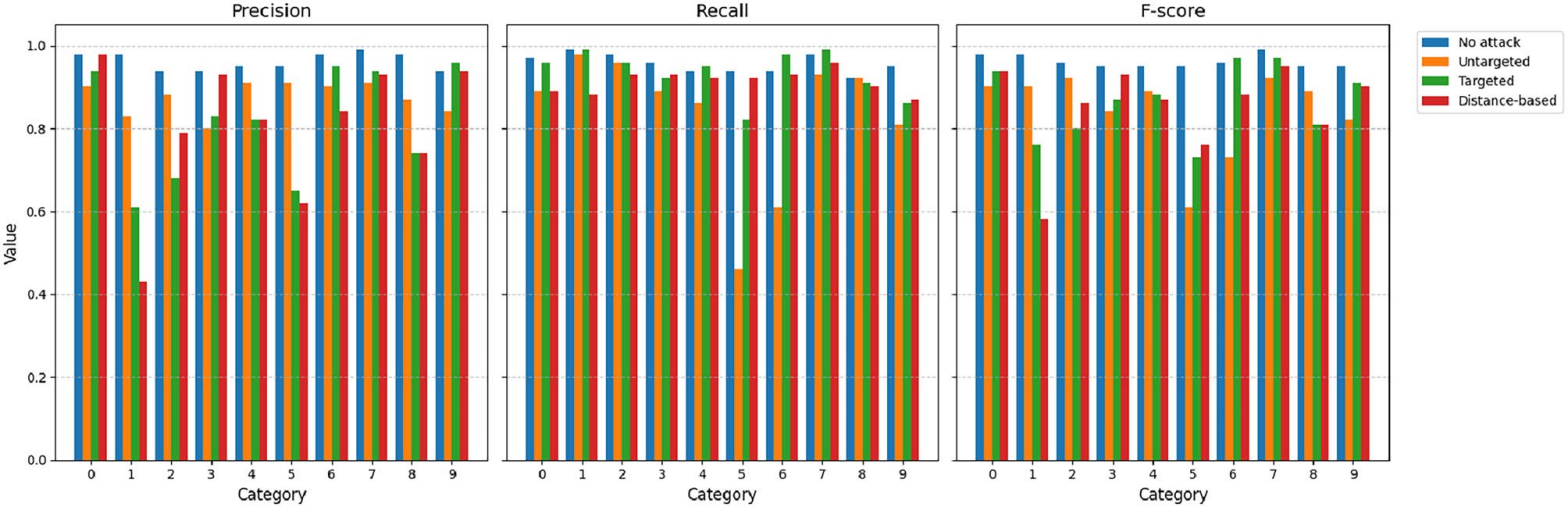
Precision, Recall, and F-score for each MNIST v1 category under different attack types (No attack, Untargeted, Targeted, Distance-based). The figure shows degraded performance under attacks, with targeted and distance-based attacks having significant impact on certain categories.

**Fig. 6:**
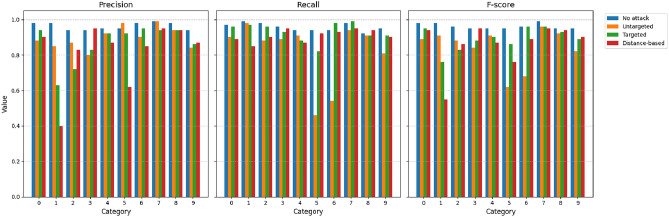
Precision, Recall, and F-score for each MNIST v2 category under different attack types (No attack, Untargeted, Targeted, Distance-based). The figure shows degraded performance under attacks, with targeted and distance-based attacks having significant impact on certain categories.

**Fig. 7:**
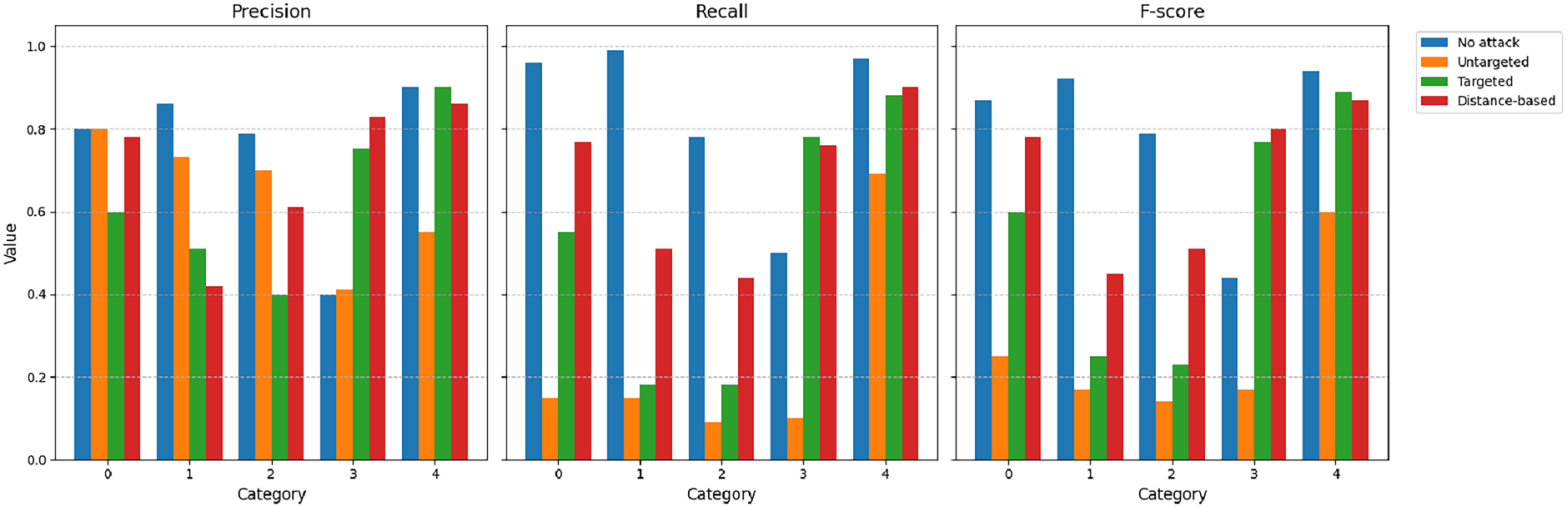
Precision, Recall, and F-score for each ECGv1 category under different attack types (No attack, Untargeted, Targeted, Distance-based). The figure shows degraded performance under attacks, with targeted and distance-based attacks having significant impact on certain categories.

**Fig. 8:**
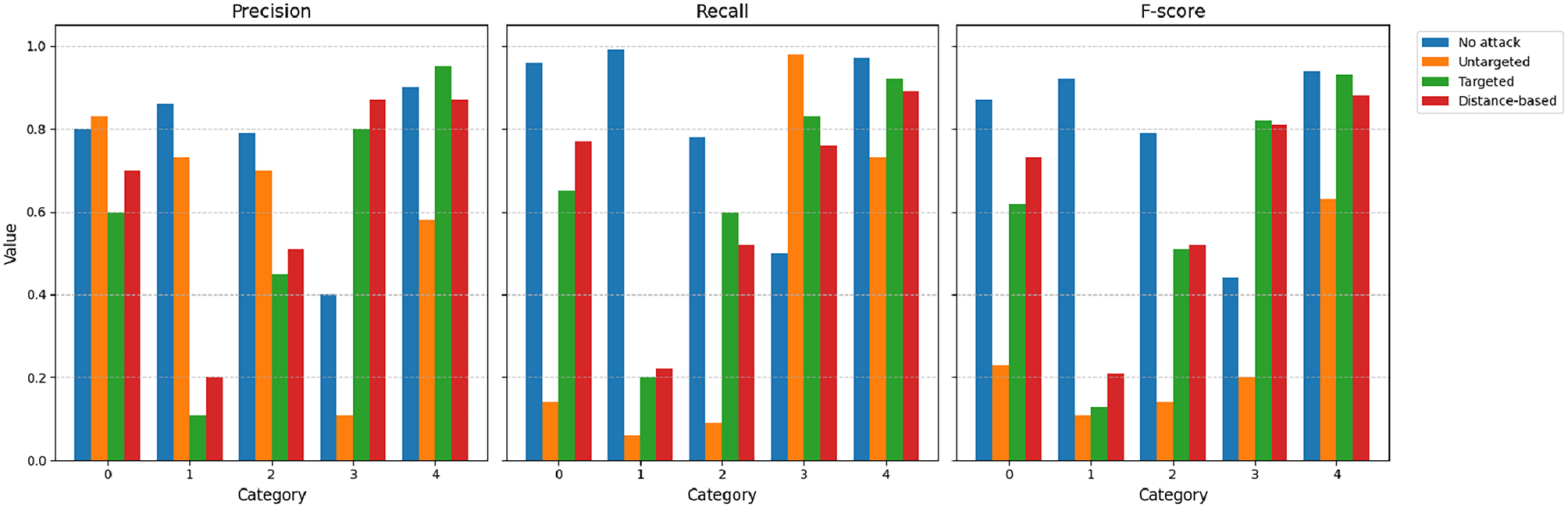
Precision, Recall, and F-score for each ECGv2 category under different attack types (No attack, Untargeted, Targeted, Distance-based). The figure shows degraded performance under attacks, with targeted and distance-based attacks having significant impact on certain categories.

We also compare the values of the precision (*P*), recall (*R*), and F-score (*F*) as provided in Figs. 5,6,7,8. The figures shows the metric values for different classes. As expected, the metrics change significantly due to the different types of proposed attacks. For example, the precision for the ECGv1 model decreases from 40% to 10% for category 1. It should be noted that ECG classification is greatly impacted by the attacks compared to the MNIST classification data. For example, the F-score is only 1% for category 2 in the ECGv2 model.

It should be noted that an attacker can adopt various strategies to affect the performance of the model according to their choice and motive. For untargeted attacks, an attacker can randomly flip labels, causing an overall reduction in accuracy and leading to low performance across all classes. In the case of targeted attacks, an attacker can set labels to specific values, biasing the model’s performance towards those labels. Similarly, for distance-based attacks, an attacker can completely reverse labels with different polarity, which would have a more severe impact on specific labels.

### Impact of changing cut layers

**Fig. 9:**
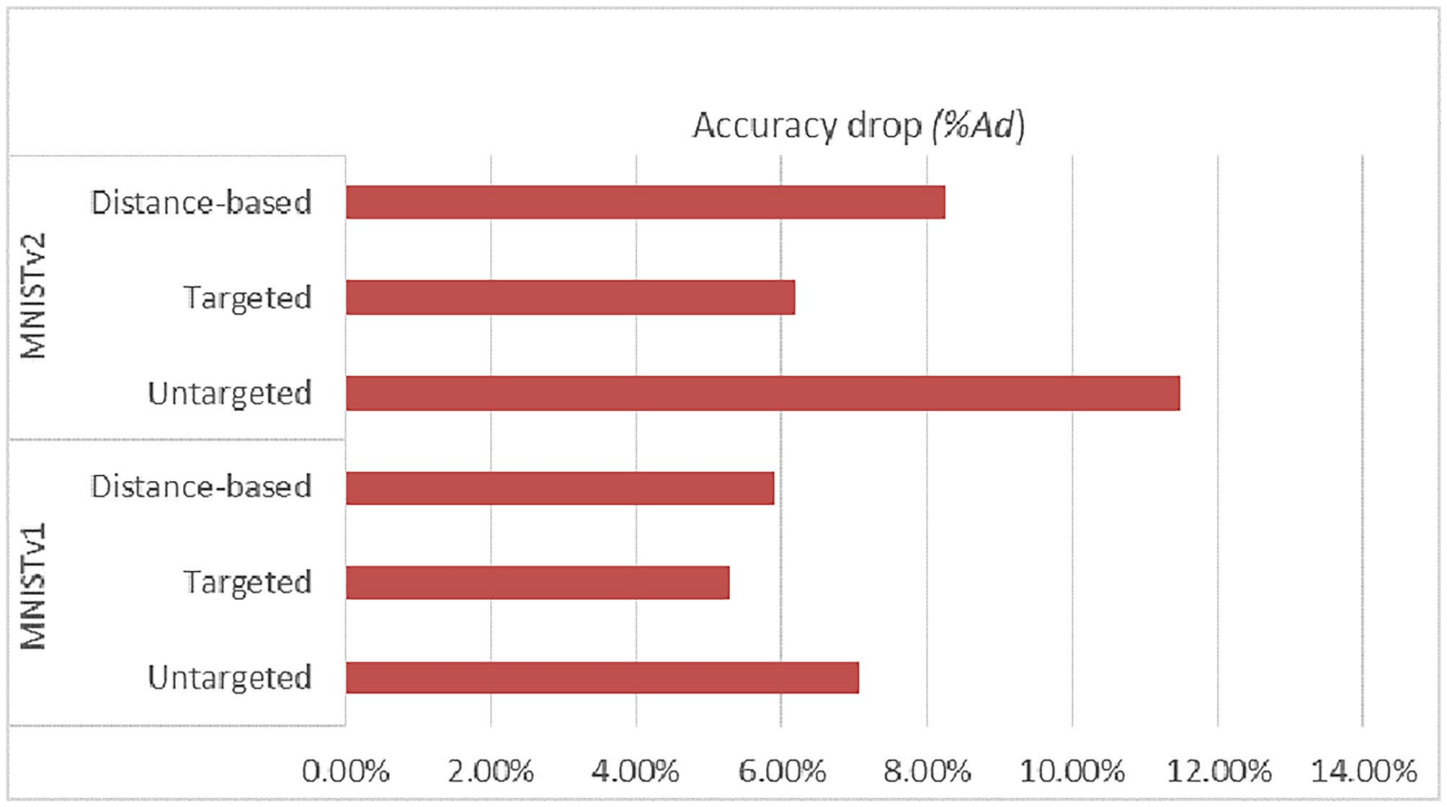
Accuracy drop $A_{d}$ v/s Split Layer in MNIST Dataset.

**Fig. 10:**
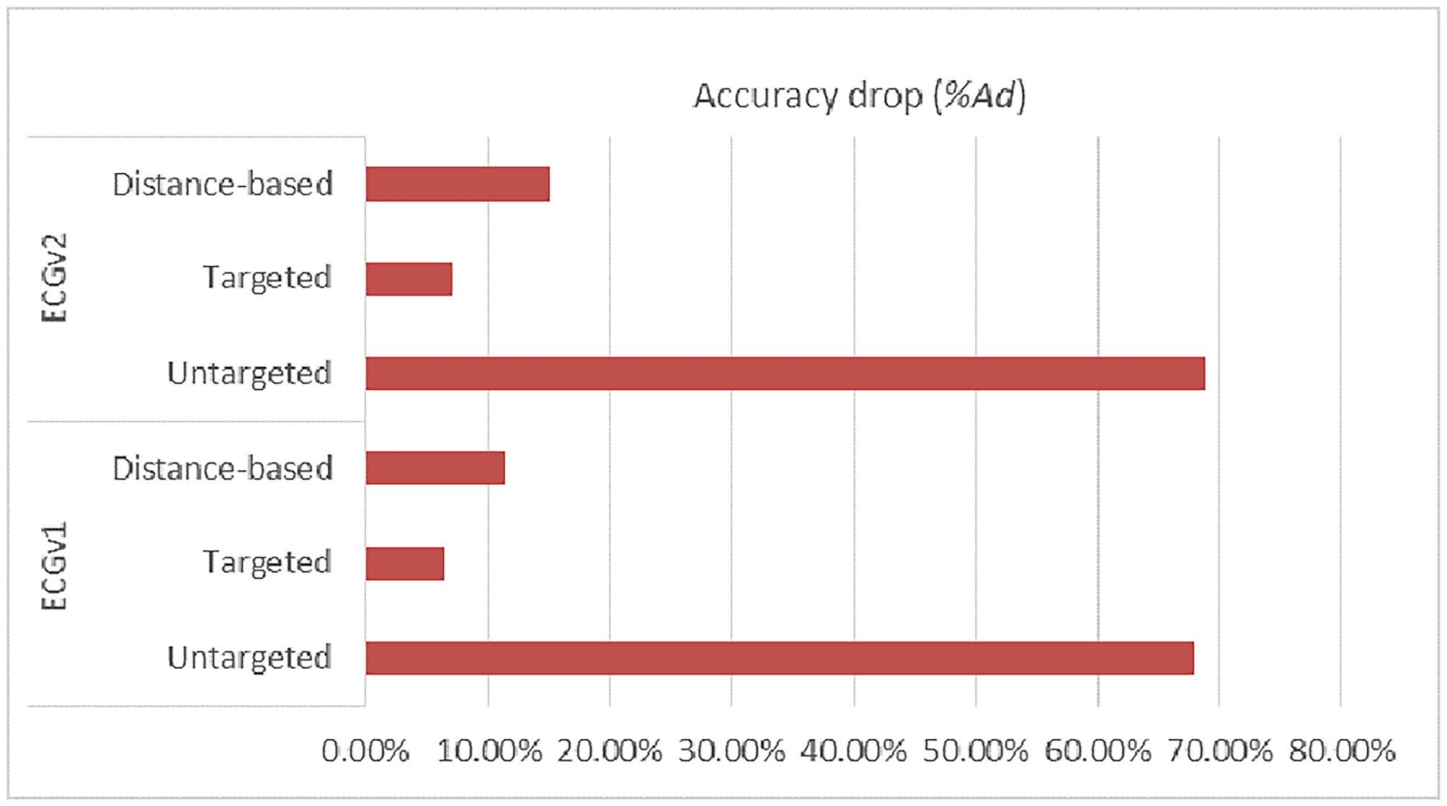
Accuracy drop $A_{d}$ v/s Split Layer in ECG Dataset.

The layer at which the model is divided between the client and server in the SFL has a serious influence on how effective poisoning attempts are. Attack intensity also varies with different cut layer choices. It is evident from the numerical data in both case studies that the poisoning attack on MNISTv2 and ECGv2 is more effective since these versions produce greater values of $A_{d}$. The reason for this is that there are now more layers in the client segment, giving the adversary greater room to initiate a more powerful and efficient attack. However, with a smaller number of model layers on the client segment, the model’s overall accuracy is not greatly affected. Figures 9 and 10 depict the relationship between accuracy drop $A_{d}$ and cut layer observed from the experimental results of the two case studies.

### Accuracy depletion with changing percentage of malicious clients

The percentage of malicious clients plays a vital role in degrading the model accuracy during data poisoning attacks. Increasing the value of malicious clients in the SFL setting can drastically reduce accuracy. Considering the possibilities of a practical scenario, it is not ideal to have a large number of malicious clients in the SFL system. In this paper, the depletion of accuracy is studied with a varied percentage of malicious clients. The results of the two case studies make it clear that even with 10% of malicious clients, the accuracy value falls to a certain level. In untargeted attacks, the higher the percentage of malicious clients, the higher the value of accuracy drops. 40% of malicious clients in ECGv2 causes the accuracy to drop from 88.89% to 26.50%. As expected, the results of all three attack strategies clearly conclude that increasing the percentage of malicious clients contributes to the success of data poisoning attacks.

After the critical analysis of experimental results, untargeted attacks have a significant impact on the classifier results. However, an attacker can still adopt targeted or distance-based attacks to reduce the classifier performance for a specific class. By adopting this strategy, it is possible to initiate attacks that cannot be directly detected while still maintaining better accuracy. This can degrade the classifier performance for one specific class chosen by the adversary.

### Defense method

To defend against the proposed label-flipping attacks, various methods can be adopted.

First, the activations that are being sent to the server can be analyzed using anomaly detection methods. If the activations from a specific set of clients differ significantly, then that client can be discarded and marked as flagged. Some of the anomaly detection methods that can be used include M-estimators and the Extreme Studentized Deviate, as they are very robust because they depend upon the central point of the data and the deviations from it^35,36^.

Another method that can be used is the clustering-based approach. The activations obtained from the different clients can be clustered together, and if some client is outside the cluster, they can be discarded or neglected at the server. Since the number of neurons in the split layer can be large, it is better to reduce the dimensionality of the intermediate activations using methods like Principal Component Analysis before performing clustering^37^.

Additionally, robust and advanced aggregation methods for activations can be used at the server instead of averaging them. Robust aggregation methods have been widely used in the context of Federated Learning to detect malicious clients^38^. However, SplitFed Learning is different because the server does not have access to the full model, but only a part of it. Nonetheless, FedServer can be used for robust aggregation. Also, the activation values that are being received by the server can be aggregated in a robust manner.

## Conclusions and future work

This paper investigates the effectiveness of various types of data poisoning attacks against Split Federated Learning (SFL). The performance of the attack strategies is evaluated under several factors, such as the number of split layers between the client and server and varying percentages of malicious clients in the SFL setting. An important indicator that reflects how accuracy decreases with attack intensity is the accuracy drop value.

Distance-based data poisoning attacks demonstrate higher efficacy than targeted attacks. The highest observed accuracy drop resulting from a distance-based attack is 8.26% for the MNIST dataset and 15.11% for the ECG dataset. Furthermore, it can be concluded that SFL is more vulnerable to untargeted attacks, which degrade the overall performance of the classifier. Additionally, SFL is more susceptible to distance-based data poisoning attacks compared to conventional targeted poisoning attacks.

It should be noted that an attacker can employ targeted or distance-based attacks to lower the performance of the classifier for a particular class, launching attacks that are difficult to detect immediately yet maintain high overall accuracy. As a result, the classifier’s performance for a selected class is degraded. This research highlights the risks and vulnerabilities of SFL, based on empirical results obtained after inducing data poisoning attacks with malicious clients.

In the future, we plan to analyze attack strategies for more complex datasets, such as colored images, videos, and text. We will begin by researching optimal split points for two-dimensional network architectures like CNNs, followed by testing new attack strategies. Research can also be conducted on the formal analysis of various defense strategies using mathematical proofs. We also intend to study attack and defense strategies where the server may be dishonest or byzantine attacks^39^. Furthermore, the use of M-estimators and Extreme Studentized Deviate (ESD) to defend against model poisoning attacks can also be explored^35,36^.

## Data Availability

This study utilized publicly available datasets. The MNIST dataset can be accessed at https://docs.ultralytics.com/datasets/classify/mnist/. The MIT-BIH Arrhythmia Database is available at https://physionet.org/content/mitdb/1.0.0/. No new datasets were generated or analyzed in the course of this study.
